# Trophic network architecture of root-associated bacterial communities determines pathogen invasion and plant health

**DOI:** 10.1038/ncomms9413

**Published:** 2015-09-24

**Authors:** Zhong Wei, Tianjie Yang, Ville-Petri Friman, Yangchun Xu, Qirong Shen, Alexandre Jousset

**Affiliations:** 1 Jiangsu Provincial Key Lab for Organic Solid Waste Utilization, National Engineering Research Center for Organic-based Fertilizers, Nanjing Agricultural University, Weigang 1, Nanjing 210095, China; 2 Imperial College London, Silwood Park Campus, Buckhurst Road, Ascot, Berkshire SL5 7PY, UK; 3 Department of Biology, University of York, Wentworth Way, York YO10 5DD, UK; 4 Institute for Environmental Biology, Ecology & Biodiversity, Utrecht University, Padualaan 8, 3584CH Utrecht, The Netherlands

## Abstract

Host-associated bacterial communities can function as an important line of defence against pathogens in animals and plants. Empirical evidence and theoretical predictions suggest that species-rich communities are more resistant to pathogen invasions. Yet, the underlying mechanisms are unclear. Here, we experimentally test how the underlying resource competition networks of resident bacterial communities affect invasion resistance to the plant pathogen *Ralstonia solanacearum* in microcosms and in tomato plant rhizosphere. We find that bipartite resource competition networks are better predictors of invasion resistance compared with resident community diversity. Specifically, communities with a combination of stabilizing configurations (low nestedness and high connectance), and a clear niche overlap with the pathogen, reduce pathogen invasion success, constrain pathogen growth within invaded communities and have lower levels of diseased plants in greenhouse experiments. Bacterial resource competition network characteristics can thus be important in explaining positive diversity–invasion resistance relationships in bacterial rhizosphere communities.

## Abstract (web summary)

Species-rich communities are thought to be more resistant to pathogen invasion. Here, Wei *et al.* show that the resource competition network architecture of root-associated bacterial communities are better predictors of plant pathogen invasion resistance than measures of community diversity.

## Graphical abstract

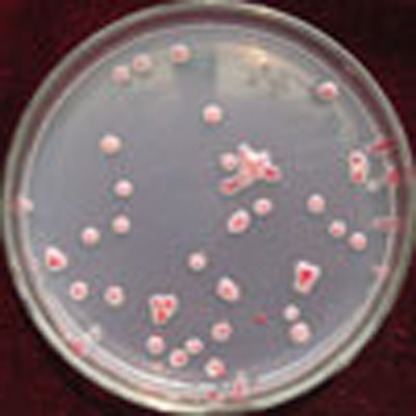

Bacterial communities cover virtually all host surfaces from skin, gut and mucosa of animals^1^,^2^ to plant roots and leaves^3^ or fungal hyphae^4^ and can function as a first line of defence against invading pathogens^5^,^6^,^7^,^8^. While it has been found that highly diverse bacterial communities are often more resistant to pathogen invasions, having positive effects on associated host health^9^,^10^, the underlying mechanisms are still debated^8^,^10^. The diversity resistance hypothesis argues that diverse communities are highly resistant to invasions due to high number of species interactions^11^ and intensified competition for niche space^9^,^12^. While this pattern is supported by both theory^11^ and experiments^9^,^13^,^14^,^15^,^16^, diversity–invasion resistance relationships are more complex^17^ and can vary from positive to negative depending on the spatial scale^18^,^19^ and resource heterogeneity^12^. As a result, identifying underlying community properties that correlate with high species diversity could be important in explaining bacterial community resistance to pathogen invasions and help to develop synthetic communities that can efficiently suppress diseases.

High diversity might increase community invasion resistance due to interactive effects on community stability^20^. For example, functionally diverse communities can be more resistant to invasions because some trophic interactions stabilize otherwise dominant competitive interactions within the community^21^. Diversity could also affect the antagonistic and facilitative trophic interaction networks between the different members of the resident community^22^,^23^,^24^. The structure of these kind of ecological networks, that is, the configuration and the distribution of the different links among interacting species, can provide strong predictions on the function and stability of ecosystems^22^. For instance, nestedness and connectance of species interaction networks can affect community stability and persistence^22^,^25^, and recent modelling studies have linked these interaction network structures to community invasion resistance^26^,^27^,^28^. As a result, interactions both within plant-associated rhizosphere communities and between the resident communities and the invading pathogen are likely to be important for plant health and fitness.

Here we directly examined the distribution of trophic links as underlying mechanisms to predict invasion resistance of plant root-associated bacterial communities against an invading pathogen and the subsequent reduction of disease incidence. We focused on the important soil-borne bacterial pathogen *Ralstonia solanacearum*, the causative agent of bacterial wilt capable of infecting over 200 different plant species across 50 different families^29^. To cause disease, *R. solanacearum* pathogen must first invade the resident bacterial communities and reach a threshold density to become virulent^30^. We specifically focused on bacterial resource competition networks as a putative mechanism linking resident community composition, pathogen invasion success and subsequent spread of bacterial wilt disease in tomato plants^9^.

Resident bacterial communities were constructed by using five, phylogenetically related but non-virulent, *Ralstonia* spp. species^31^, which were originally isolated from the tomato plant rhizosphere. The rationale behind using closely related but non-virulent species was to maximize the similarity in resource use between the resident community and the invading pathogen via close phylogenetic relationship^31^. We first verified that the tested species did not show any direct antagonism towards each other in terms of growth inhibition^32^, that is, that these species interacted only via resource competition. We then characterized the resource consumption patterns of both the pathogen and each of the resident species on 48 labile carbon sources typically found in tomato plant rhizosphere (amino acids, organic acids and sugars). This information was then used to build bipartite networks reflecting bacterial interactions mediated by resource competition (Fig. 1). In this approach all trophic links, measured by the ability of each consumer (bacteria) to grow on each resource (single carbon source) are summarized in matrices to characterize network properties of different communities. By using different combinations of bacterial species with varying species richness and community composition (31 different communities covering all possible combination of the five resident community species), we could define the niche overlap in resource competition patterns for the invading pathogen and each resident community, as well as the connectance and nestedness of the resource competition networks of the resident communities, chosen for their relevance for resource competition and prevalence in nature^33^ (Fig. 1).

Three different network variables were used to describe (1) similarity in resource consumption between the resident community and the invader (niche overlap) and (2) the intensity of resource competition within resident communities (connectance and nestedness). In our study, niche overlap describes the ecological similarity between the invader and the resident community respective to their resource use. High niche overlap between the resident community and the invader is thus likely to be a decisive factor affecting invasion success^34^,^35^ as high overlap will increase resource competition and the probability of competitive exclusion of the invader. In our study, connectance describes the proportion of realized resource–consumer interactions from all possible resource–consumer interactions, and is thus reflective of the average niche breadth of the resident bacterial community. In highly connected communities all species of the resident community are able to use resources similarly, which could increase the consumption of every individual resource (Fig. 1). High connectance could thus decrease pathogen invasion success via reduced opportunities for pathogen growth. While previous modelling work has shown that high connectance could either constrain^26^ or promote^27^ invasions depending on the relationship between species richness and connectance, food webs containing consumers with very diverse diet breadths (generalists) have been found to be more resistant to invasions^27^. High connectance is thus likely to constrain pathogen invasion since all members of the resident community will efficiently exploit most resources. In general, nestedness describes trophic interactions between generalist and specialist resource consumers within the community. In highly nested networks, generalist species will have many resource–consumer links, which are shared by specialist species that have only few resource–consumer links (Fig. 1). In mutualistic pollinator networks, nestedness has been shown to stabilize communities and enhance persistence (the number of species coexisting at equilibrium)^22^,^25^. However, in resource competition networks nestedness is likely to have a destabilizing effect^22^ because specialists need to compete for subsets of resources that are used also by generalists, which will likely lead to decreased ranges of species' growth rates. As a result, communities with nested resource competition networks could be less stable and less even, and thus, more susceptible to invasions.

To test these hypotheses, we conducted replicated invasion experiments in laboratory microcosms (in 96-well microtiter plates) and in the tomato plant soil (rhizosphere) to link resident community composition, invasion resistance and spread of bacterial wilt plant disease. Our results show that bacterial resource competition networks are better at predicting invasion resistance than community diversity. Specifically, resident communities with stabilizing configurations (low nestedness and high connectance), and a clear niche overlap with the pathogen, reduce pathogen invasion success very efficiently leading to lowest levels of bacterial wilt plant disease. Bacterial resource competition network characteristics can thus be important in explaining positive diversity–invasion resistance relationships in bacterial rhizosphere communities.

## Results

### Pathogen invasion success in laboratory microcosms

**Figure 2:**
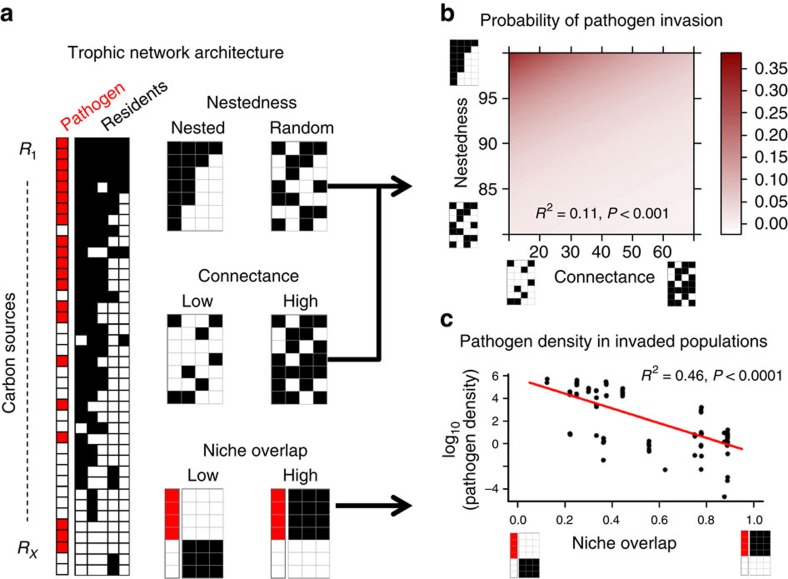
Pathogen invasion success measured in microcosm experiments. (**a**) A schematic matrix capturing resource competition interactions between the pathogen (red boxes) and resident community species (black boxes); filled squares indicate that the given bacteria consume a given resource. (**b**) Pathogen invasion success (probability of invader establishment, visualized as heatmap showing the results of the used GLM) was lowest in non-nested and highly connected resident communities. (**c**) Pathogen growth in successfully invaded communities was constrained most when the resident communities had high niche overlap with the pathogen.

Increasing resident community diversity reduced pathogen invasion success, defined as the portability of successful invasion (Table 1). At the level of resource competition networks, resident communities with low nestedness and high connectance constrained pathogen invasion most efficiently (*R*^2^=0.11, the effect of niche overlap non-significant, Fig. 2; Table 1). Similar to pathogen invasion success, high resident community diversity reduced pathogen growth within successfully invaded communities (Table 1). At the network level, pathogen growth within successfully invaded communities was constrained only by high niche overlap in resource competition patterns between the invading pathogen and resident community (*R*^2^=0.46, Fig. 2; the effects of nestedness and connectance non-significant, Table 1). Crucially, the network-based model was a better predictor of both pathogen invasion success and subsequent growth within invaded resident communities than the diversity-based model.

Together these results suggest that the characteristics of bacterial resource competition networks could partially explain why more diverse communities were often resistant to pathogen invasions: while nestedness and connectance were important for predicting pathogen invasion probability, high niche overlap was the best predictor for subsequent pathogen growth within successfully invaded resident communities.

### Pathogen invasion success in tomato plant rhizosphere

**Figure 3:**
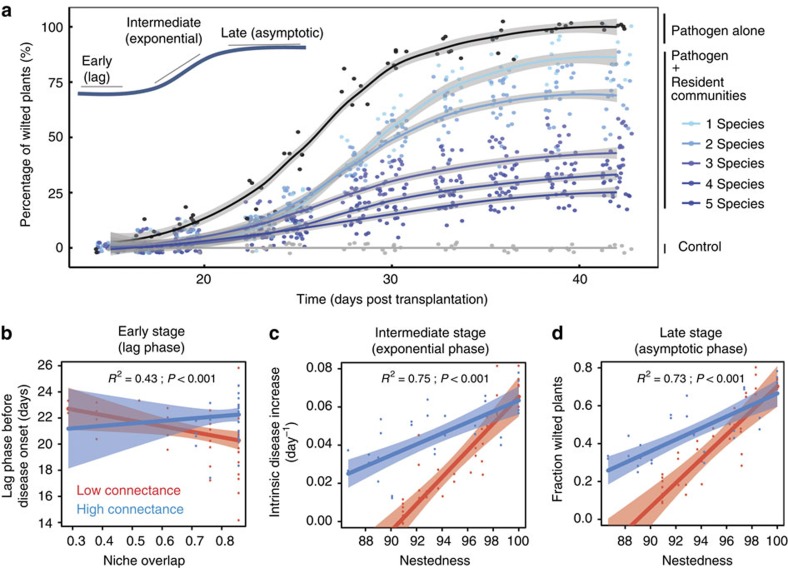
Pathogen invasion success measured in tomato plant rhizosphere. (**a**) Spread of bacterial wilt plant disease in the absence and presence of resident communities (control denotes for treatment without any bacteria). Disease spread was fitted with the data by using a logistic regression to obtain three variables describing the dynamics of disease dynamics: lag time before disease onset (early stage), the exponential rate of disease spread (intermediate stage) and the asymptotic disease saturation (late stage). (**b**–**d**) Main effects and interactions between connectance, niche overlap and nestedness on disease development during each stage of infection. The *R*^*2*^ and *P* values refer to the most parsimonious model fitted for each disease stage.

The proportion of wilted plants (clear symptom of *Ralstonia* infection, which correlates with pathogen density in the rhizosphere and thus with invasion success^32^) increased with time and the bacterial wilt disease spread fitted well with a logistic regression (Fig. 3; Table 1). Bacterial wilt disease incidence rapidly increased after a 3-week initial lag phase before reaching equilibrium. The presence of resident communities reduced disease spread and this effect varied considerably between different communities. Increasing resident community diversity reduced the risk of tomato plant wilting at early, intermediate and late stages of infection (Fig. 3; Table 1). Interestingly, the same network variables that predicted resident community resistance to pathogen invasion in microcosm experiments were also important in explaining bacterial wilt spread in tomato plant rhizosphere. During the early stage of infection, resident communities with low nestedness, high connectance and high niche overlap with the pathogen, delayed the spread of bacterial wilt; in other words, the onset of disease spread took longer (*R*^2^=0.43, Table 1; Fig. 3). These relationships persisted throughout the rest of the invasion experiment, the network model being the best predictor of disease spread during the intermediate (*R*^2^=0.75, Table 1; Fig. 3) and late infection stages (*R*^2^=0.73, Table 1; Fig. 3). Note that high connectance promoted disease spread during the intermediate stage of infection before becoming non-significant during the late stage of infection (*R*^2^=0.75, Table 1; Fig. 3). We also found that network variables had interactive effects on disease spread. In general, nestedness and connectance reduced each other's effects of at every stage of infection (Table 1). Similarly, high niche overlap reduced the effect of nestedness during the late stage of infection, while niche overlap and connectance strengthened each other's effects during the early stage of infection (Table 1; correlated effects are analysed in more detail in the next chapter). Alike to laboratory invasion experiments, the network-based model was a better predictor of tomato plant wilting compared with the diversity-based model (Table 1).

These results suggest that the same network variables that predicted pathogen invasion success in simple laboratory microcosms can also predict pathogen invasion success in more complicated tomato plant rhizosphere. Low nestedness and high niche overlap were especially important in reducing the spread of bacterial wilt during the intermediate and late stages of pathogen invasion. While network variables had interactive effects on disease spread, their relative importance was generally smaller compared with main effects of network variables.

### Linking diversity and networks for invasion resistance

**Figure 4:**
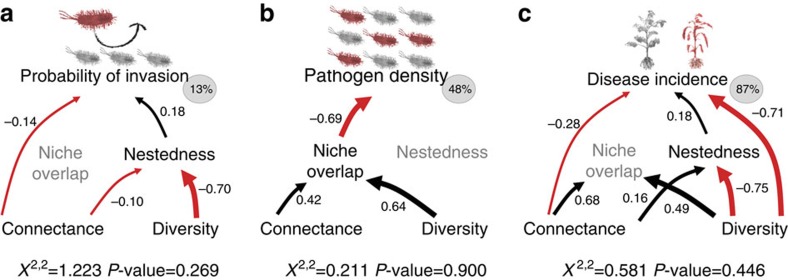
Structural equation models linking diversity and network variables with pathogen invasion success and subsequent spread of bacterial wilt plant disease. (**a**,**b**) Results from microcosm invasion experiments (probability of invasion and pathogen density in invaded communities). (**c**) Results from rhizosphere invasion experiment (disease spread during the late stage of the infection). Nestedness, connectance and niche overlap with the pathogen explained most of the invasion process in microcosm invasion experiments. In the rhizosphere invasion experiment, the direct effect of diversity was important for bacterial wilt disease spread. Grey circles left of each panel denote for the proportion of the total variance explained and the numbers on the arrows denote standardized correlation coefficients. Red arrows denote for negative effect on invasion process by the high value of the given variable, and black arrows denote for positive effect on invasion process by the high value of the given variable; arrow widths correspond with the relative effect size of each variable.

We used structural equation modelling (path analysis) to describe the directed dependencies among diversity and different network variables. We first concentrated on pathogen invasion success in microtitre plate invasions assays. Only high community connectance had direct negative effect on pathogen invasion success (Fig. 4a). The effects of diversity and connectance were further channelled indirectly into reduction in resident community nestedness, which then directly constrained pathogen invasion success (Fig. 4a). Consistent with the generalized linear models (GLMs) on microtitre plate invasion, the effect of niche overlap was non-significant. We next focused on pathogen growth in successfully invaded microcosm communities (Fig. 4b). The significant effects of diversity and connectance were channeled into increase in niche overlap, which led to reduction in the pathogen growth (Fig. 4b). Consistent with the GLM approach, the effect of nestedness was non-significant. Finally, we compared the relative importance of diversity and network variables of resident community for tomato plant survival. Both high connectance and high diversity directly reduced the levels of bacterial wilt disease (Fig. 4c). Moreover, both connectance and diversity had indirect effects on disease spread via nestedness (connectance increased and diversity decreased), which directly constrained disease spread (Fig. 4c). High connectance and diversity also indirectly increased the niche overlap with the pathogen. Even though retained in the final model, the effect of niche overlap itself was non-significant (Fig. 4c).

In conclusion, network variables were better predictors of resident community invasion resistance in microcosm experiments compared with diversity. However, this pattern was altered in the rhizosphere invasion experiments, where the mix of both indirect and direct effects of diversity and network variables best explained the pathogen invasion success and subsequent spread of tomato bacterial wilt disease.

## Discussion

Here we show that simple bipartite resource competition networks^33^ can reliably predict resident bacterial community invasion resistance to the plant pathogen *R. solanacearum*. Biodiversity of microbial communities is increasingly recognized to constrain invasion by pathogens^9^,^36^. In the present study we concentrate on underlying mechanisms and show that the structure of resource competition networks provide a missing link between community composition and pathogen invasion. We show that resident community invasion resistance and its subsequent ability to prevent tomato bacterial wilt disease could be explained by using only few key resource competition network variables: connectance, nestedness and niche overlap with the pathogen. Crucially, network-based models had consistently better explanatory power compared with diversity-based models (Table 1). This suggests that diversity–invasion resistance relationships could be mechanistically explained by the underlying interaction network architecture. Even though the network variables were the most important predictors of pathogen invasion success in microcosm experiment, resident community diversity also had direct effects on bacterial wilt disease spread in tomato plant rhizosphere. Both species interaction networks and species richness may thus be important for the invasion resistance of bacterial communities.

We hypothesized that pathogen invasion success should decrease with increasing niche overlap between the resident communities and invading pathogen due to intensified resource competition. While niche overlap had no effect on pathogen invasion probability in microcosm experiments, it constrained subsequent pathogen multiplication in successfully invaded resident communities. This result is in line with previous studies reporting that high catabolic similarity between the rhizosphere community and the invading pathogen reduces the community invasibility^34^. Interestingly, the relative importance of niche overlap was low compared with the effects of nestedness and connectance in the rhizosphere invasion experiments. Together, these results suggest that invasion resistance is mediated not only by competitive exclusion of the pathogen due to similar niche breadth but is also affected by competitive interactions within the resident microbial community.

High connectance within rhizosphere communities could decrease pathogen invasion success if it leads to generally more efficient consumption of resources, and hence increased competition^9^,^11^,^12^. Alternatively, high connectance could increase pathogen invasion success if the resource competition is stronger among the members of rhizosphere community than between the members of rhizosphere community and the invading pathogen. We found that highly connected resident communities decreased the probability of pathogen invasion success in microcosm experiments. Mechanistically, this could have been due to more efficient consumption of resources by highly connected resident communities, which could have prevented pathogen establishment and subsequent growth. These patterns also fitted well to the disease spread in tomato plant rhizosphere, with the exception that connectance promoted disease spread during the intermediate stage of infection. The effects of connectance on resident community invasion resistance could depend on the spatial structure of the environment and could change during plant development. For example, it is possible that rhizosphere bacteria were able to consume resources more efficiently in spatially homogenous microcosm environment (liquid media) compared to spatially heterogeneous soil environment, where resource competition between rhizosphere community members was likely more intense at the local scale^37^,^38^. As a result, high connectance could have increased the resource competition within the rhizosphere community leading to community destabilization^39^ and successful pathogen invasion in the soil. This effect could have been stronger during the intermediate stages of infection making highly connected resident communities more susceptible for pathogen invasion. Interestingly, high connectance also reduced the negative effect of high nestedness on community invasion resistance, indicating that different network characteristics may together shape invasion resistance in rhizosphere bacterial communities.

Lastly, we predicted that low nestedness could increase resident community invasion resistance due to less intense resource competition among generalist and specialist members of resident communities. We found that highly nested resident communities were more susceptible to pathogen invasion and subsequent spread of bacterial wilt plant disease in the tomato plant rhizosphere. Nested resource competition patterns could destabilize rhizosphere communities because specialist species need to compete for a subset of the resources used by generalists^22^, which could lead to a decrease in bacterial productivity in general^40^. In contrast to mutualistic nested networks, where generalists can facilitate the coexistence of specialists^25^,^33^, generalists are likely to have negative effect on specialists via asymmetrical resource competition in highly nested exploitative interaction networks. Competition-mediated extinctions within the resident community^22^ could thus lead to less efficient utilization of resource niche space and expose resident communities to invasions. Alternatively, when community assembly is niche-driven, random resource use patterns may result in a higher community evenness^41^, which in turn could improve community resistance to invasion^42^,^43^. In this study, we used bacterial wilt disease symptoms as a proxy for both pathogen density changes in the greenhouse experiment: the prevalence of bacterial wilt correlates directly with *R. solanacearum* densities in rhizosphere^32^. Therefore, further experiments are needed to link the disease spread with changes in the density of resident species in soil.

Interestingly, diversity had direct positive effects on rhizosphere community invasion resistance in the tomato plant soil, which was not explained by resource competition network characteristics. There are multiple non-exclusive explanations for this. First, it is likely that some other factors in addition to resource competition patterns were important for resident community and pathogen fitness in the soil. For example, differences in species ability to colonize plant roots could have played significant role for the establishment of protective rhizosphere communities^30^. Moreover, some members of the resident community might have been able to induce systemic plant resistances against pathogens^44^ leading to increased invasion resistance. Unfortunately, it is impossible to disentangle these hypotheses on the basis of this data, and thus, further experiments are needed to validate these hypotheses.

Albeit being effective in demonstrating causal relationships between resident community interaction network structure, invasion resistance and subsequent disease spread in the soil, our simplified approach has some caveats that might lead to different invasion resistance patterns in more complex natural systems. Firstly, our diversity manipulation included only *Ralstonia* species that were closely related with the pathogen. As a result, resident community bacterial diversity could have different effect on invasion resistance depending on the genetic distance between the resident bacterial community and the invading pathogen. Further, our model focused only on resource-mediated competition. In a natural system, increasing bacterial diversity could also increase the strength of antagonistic interactions, which could potentially make communities more susceptible to pathogen invasion^45^. Our simplified model rhizosphere bacterial community also contained only one trophic level: the resource-consuming bacteria. Natural soil rhizosphere communities are more complex having multiple trophic levels such as parasitic phages and predatory protists and nematodes^46^. Thus, our approach could be extended in the future to a higher number of trophic levels and different types of species interactions, including antagonistic competition^47^ and cross-feeding^48^. More complex food webs could be decomposed into a subset of bipartite networks^49^ to improve the predictive power and correspondence with the more complex microbe–plant ecosystems^50^. Thirdly, the positive effects of resident bacterial communities on invasion resistance might be short-lived as rhizosphere bacterial communities, varying temporally during plant development^51^ or growth season^52^. Furthermore, bacterial interaction networks and/or community composition are likely to change after crop plant removal in agricultural systems, which could affect the rhizosphere community invasion resistance on a longer term^32^. Finally, it is possible that bacterial species embedded in resident community or the invading pathogen evolves. For example, *R. solanacearum* pathogen can rapidly evolve respective to its resource consumption patterns^53^,^54^ or host species^55^. However, it is not known if such adaptive processes could be driven or are affected by resource competition patterns with resident rhizosphere community members.

Soil-borne plant pathogens remain difficult to control because their populations are variable in time and space, and because they can often overcome plant resistance via evolution^56^. Currently an important part of global food production is lost to soil-borne plant diseases^56^. Thus, improving rhizosphere community invasion resistance could significantly improve crop yields across different agricultural systems. Here, we propose an ecological network approach to improve plant health by directly engineering the resource competition interaction networks of plant-associated bacterial communities. Instead of antagonizing the pathogen, rhizosphere invasion resistance could be optimized with interaction network structure that maximizes competition with the pathogen and simultaneously minimizes the competition within the resident bacterial communities. Even though our study is at least an order of magnitude simpler than any natural rhizosphere community, we propose that similar ecological mechanisms could predict the functioning of more complex microbiomes potentially affecting animal and human health^5^,^8^.

## Methods

### Bacterial strains and plasmids

We used *R. solanacearum* QL-Rs1115 (GenBank accession GU390462) tagged with the pYC12-mCherry plasmid as an invading bacterial pathogen^32^. *R. solanacearum* is a soil-borne, Gram-negative bacterium that invades plant roots and multiplies in the cortical tissue before invading the xylem^30^. In a matter of hours, the bacteria spread into the crown and stem, causing wilt, a generalized necrosis and plant death. Root colonization is the first step and prerequisite for plant infection^9^. As roots are densely populated with diverse bacterial communities, interactions with the resident bacterial community could affect pathogen invasion success, and ultimately, host infection^57^.

We used five non-virulent but closely related *Ralstonia* spp. strains to construct model resident bacterial communities that were exposed to pathogen invasion. All resident community bacterial strains were isolated from the tomato rhizosphere and first grown on South Africa semi selective medium (SMSA-E)^31^. Isolated *Ralstonia* colonies were identified on the basis of 16S rRNA gene sequence (27F and 1492R primers^58^), deposited in GenBank under the accession number JN699058 (strain QL-A2), KJ780056 (strain QL-A3), HQ267096 (strain QL-A6), KJ780054 (strain QL-117) and KJ780055 (strain QL-140) and were cryopreserved at −80 °C in glycerol (20% ending concentration). None of these strains were virulent and did not cause any bacterial wilt disease symptoms when injected into tomato stems^31^. Moreover, none of the resident community strains showed direct antagonism towards each other or the pathogenic *R. solanacearum* QL-Rs1115 strain on soft agar overlay essays, which suggests that these strains interact indirectly via resource competition. All bacterial strains and plasmids are listed in supplementary Table 1.

### Construction of resident bacterial communities

We used a full factorial design to construct model resident bacterial communities covering all possible species richness and community compositions (31 communities in total; Supplementary Table 2). Resident bacterial communities were then subjected to invasion by the pathogenic *R. solanacearum* QL-Rs1115 strain first in microtiter plates in simplified laboratory conditions, and second, in tomato plant rhizosphere in over 1 month long greenhouse experiment. We used a substitutive design in all experiments, meaning that the initial resident community densities were adjusted to the same level in all community composition treatments^59^.

### Characterization of bacterial resource competition networks

We used bacterial resource consumption patterns to determine indirect resource competition networks (1) between different members of resident communities and (2) between different resident communities and the invading pathogen. To this end, we first characterized the resource consumption pattern of each bacterial strain individually in single-species monocultures on 48 different single carbon resources representative of tomato root exudates. Briefly, overnight cultures were adjusted to an optical density (OD_600_) of 0.1 and grown in microtitre plates in 150 μl OS minimal medium^60^ supplemented with 48 compounds representative of amino acids, organic acids and sugars found in tomato root exudates (Supplementary Table 3). After 48 h growth at 25 °C with agitation, optical density was recorded in a SpectraMax M5 Plate reader (Molecular Devices, Sunnyvale, CA, USA) and wells with an OD_600_>0.05 were scored as positive growth on given substrate. We then used these single-species and one-resource interactions to determine resource consumption patterns (1) between the different members of resident communities and (2) between different resident communities and the invading pathogen. We first defined niche overlap between resident communities and the invading pathogen as the proportion of resources used by the pathogen and by at least one member of the given resident community^34^. We the estimated two network variables that have been reported to be important for biological invasions^26^,^27^,^28^ for all resident communities: connectance and nestedness. In our system, connectance describes the proportion of realized trophic interactions from all possible trophic interactions and is thus reflective of species diet breadth: in highly connected communities all species would be able to use all resources while in poorly connected communities species use different subset of resources. Connectance was estimated as a proportion of realized links between species and resources they share in all given communities by using the formula *C*=*L*/*S*^2^, where L denotes for links between species (number of resources all species can consume) and *S* denotes for species richness of the community. Nestedness describes the trophic interactions between generalist and specialist resource consumers. In highly nested networks, generalist species have many links, which are shared by specialist species that have only few links. Mathematically, nestedness was estimated by the system's ‘temperature' via binary matrixes in three steps by computing first an isocline of perfect order, reorganizing the matrix by permuting rows and columns in the way that maximizes its nestedness and comparing its normalized distance to the isocline^61^. The temperature of the matrix is the sum of these distances, normalized in such a way that it ranges between 0 for a perfectly unnested (random) matrix and 100 for a maximally ‘nested' matrix. Nestedness was computed with the function nested() of the R package bipartite using the binmatnest2 algorithm.

### Pathogen invasion success in laboratory microcosms

All species of resident bacterial communities were first grown alone in liquid nutrient medium (NA, glucose 10.0 g l^−1^, peptone 5.0 g l^−1^, yeast extract 0.5 g l^−1^, beef extract 3.0 g l^−1^, pH 7.0) on a shaker at 170 r.p.m., 30 °C for 12 h, after which 31 resident communities were constructed with a starting density of 10^7^ cells per ml. All resident communities were inoculated to 20 different resources environments (total of 620 individual populations), each of which were composed of OS minimal medium supplemented with a random mixture of 20 carbons selected from the total pool of 48 resources (supplementary table 4). As a result, the network variables (connectance, nestedness and niche overlap between resident community and pathogen) varied depending on the resource environment. Model resident communities were then subsequently exposed to invasion by mCherry-tagged *R. solanacearum* QL-Rs1115 strain (10^5^ cells per ml) or grown alone in the absence of pathogen in 96 wells microtitre plates (200 μl per well) at 30 °C with agitation. Resident community growth was recorded after 48 h on the basis of optical density (OD_600_) and pathogen growth on the basis of the mCherry fluorescence signal (excitation: 587 nm, emission: 610 nm). The invasion process was broken into two steps. First, the probability of successful invasion was measured as a binary variable for each resident community resource environment combination: invasion was scored as successful if the fluorescence signal was higher, and unsuccessful if it was lower, than the background signal of the corresponding control treatment. Second, we quantified the pathogen invasion success within successfully invaded resident communities as pathogen density based on the level of fluorescence signal (bacterial autofluorescence signal of corresponding control treatment were used as a blank^62^).

### Disease spread in tomato

The spread of *Ralstonia* wilt in tomato plants previously inoculated with the different resident bacterial communities was tested in a 42 days long greenhouse experiment (Supplementary Fig. 1). As disease incidence is directly correlated to pathogen density around plant roots^31^, we used wilting symptoms as proxy for following pathogen invasion of rhizosphere communities. Similar to microcosm invasion experiments, we used the same 31 non-virulent resident communities (two replicates each), a positive control containing only the pathogen (three replicates) and a negative control without any bacteria (three replicates). Surface sterilized tomato seeds (*Lycopersicon esculentum,* cultivar ‘*Jiangshu*') were germinated on water-agar plates for three days before sowing into seedling plates containing Cobalt -60-sterilized seedling substrate (Huainong, Huaian soil and fertilizer Institute, Huaian, China). Tomato plants at the three-leaf stage (11 days after sowing) were transplanted to seedling trays (Supplementary Fig. 1) containing the same seedling substrate as describe above and inoculated with resident bacterial communities by drenching method (ending concentration of 10^8^ CFU per g soil)^31^. The invasion experiment was initiated 1 week later by inoculating resident bacterial communities with the pathogen (ending concentration of 10^6^ CFU per g soil). Tomato plants were grown for 42 days after transplantation in a greenhouse with natural temperature variation ranging from 25 to 35 °C and watered with sterile water regularly. Note that the plants from individual single seedling plates were not considered as independent replicates, but instead the number of wilted plants per seedling plate was used as a disease index. Seedling plates were arranged in randomized order and rearranged randomly every 2 days. The number of wilted plants per seedling plate was recorded at daily basis after 17th day post transplantation (no symptoms were observed before day 17). As we were unable to quantify available carbon resources in the rhizosphere directly, we made the assumption that all 48 resources used to characterize bacterial resource consumption patterns were available in the rhizosphere.

### Statistical analyses

Pathogen invasion pattern in microcosm experiment was analysed with GLM assuming binomial data distribution for invasion success analysis and lognormal data distribution for invader density increase analysis. For greenhouse invasions experiments, disease spread over time was assessed as a logistic growth curve spanning the percentage of wilted plants as a function of time during whole duration of the experiment. The curves were fitted individually for each tray, in an approach very similar to mortality curves. This fit produced three variables: the lag phase (days after pathogen addition) before disease onset, the intrinsic increase of wilted plants (day^−1^) and the asymptotic disease prevalence (% of wilted plants); later referred to as early, intermediate and late stages of infection, respectively. Curves were fitted individually for each tray—an approach very similar to survival analysis—and the resulting variables used as dependent variables in independent GLMs, weighted by –log_10_(SE) of the parameter estimates to account for the quality of the curve fitting. Due to potential correlation between connectance and nestedness, sequential analysis was used to uncover the most parsimonious GLM's. To this end, we used stepwise model selection using the Akaike information criteria (AIC) to select the model with best explanatory power (step() function in R). We used both a backward elimination (starting with a full model including nestedness, connectance and niche overlap and their first-order interactions) and forward-selection model (from simple to more complex) to avoid selecting a local AIC minimum^63^. The explanatory power of network models was further compared with classical biodiversity-ecosystem functioning models, which explained pathogen invasion success and disease spread as a function of diversity (continuous) or the presence-absence of each species.

Finally, we used structural equation modelling approach to study the direct and indirect effects of diversity and resource competition network variables for the probability of pathogen invasion and growth in microcosm invasion experiments, and for the asymptotic spread of bacterial wilt in the greenhouse invasion experiment. Briefly, we based our approach on a recent work^22^ and used diversity and connectance of the resident community as exogenous variables and nestedness and niche overlap as endogenous variables to disentangle the direct and indirect effects of each variable on pathogen invasion success and spread of bacterial wilt disease. Please note that the structural equation modelling approach does not allow us to include interactions into the model, and thus the results might slightly differ from the models presented in Table 1. The most parsimonious model was derived from the full model by removing non-significant pathways using Bayesian information criterion and chi-square tests. s.e. of means was computed with the lavaan package in R 3.0.3.

## Additional information

**How to cite this article**: Wei, Z. *et al.* Trophic network architecture of root-associated bacterial communities determines pathogen invasion and plant health. *Nat. Commun.* 6:8413 doi: 10.1038/ncomms9413 (2015).

## Supplementary Material

Supplementary Information Supplementary Figure 1, Supplementary Table 1-4 and Supplementary References

## Figures and Tables

**Figure 1:**
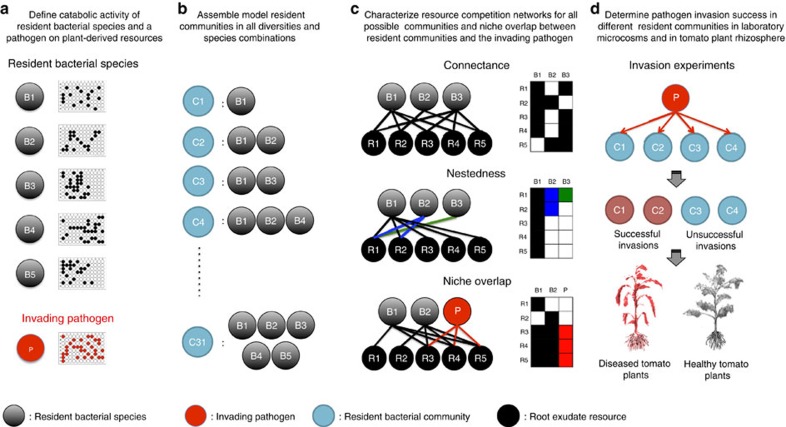
Conceptual framework and experimental design. We first characterized resource consumption patterns for both resident community species and the invading pathogen on carbon sources representative for conditions prevailing around tomato roots (**a**). We then assembled resident communities in all possible species combinations (**b**), and defined resource competition networks characteristics (connectance, nestedness and niche overlap) for all assembled communities. Filled squares denote consumed and white squares unconsumed resource, respectively. (**c**). Finally, every assembled community was exposed to pathogen invasion in laboratory microcosms and tomato plant rhizosphere to link resident community invasion resistance with network characteristics (**d**). P denotes for pathogen, B1–B5 denotes different resident community members, C1–C31 denotes different possible resident community compositions and R1–R5 denotes different tomato root exudate environments.

**Table 1:** Comparison of bipartite network-based and diversity-based general linear mixed models (GLM) for pathogen invasion success in microcosms and rhizosphere.

	**d.f.**	***In vitro*** **assays**						**Disease spread in greenhouse**								
		**Probability of invasion**			**Invader density**			**Early (lag) phase**			**Intermediate (exponential) phase)**			**Late (assymptotic) phase**		
			**F**	***P*****-value**		**F**	***P*****-value**		**F**	***P*****-value**		**F**	***P*****-value**		**F**	***P*****-value**
*Species richness*																
Species richness	1	↓	8.4	0.004	↓	18.3	<0.0001	↓	18.75	<0.0001	↓	151.5	<0.0001	↓	122.9	<0.0001
Model summary			*R*^2^: 0.02	AIC: 389.8		*R*^2^: 0.23	AIC:265.5		*R*^2^: 0.23	AIC: 244.4		*R*^2^:0.72	AIC: −356.6		*R*^2^: 067	AIC: −77.44
																
*Networks metrics*																
Nestedness (NE)	1	↑	19.6	<0.0001		Not retained		↓	5.03	0.03	↑	139.34	<0.0001	↑	119.1	<0.0001
Niche overlap (NO)	1		Not retained		↓	49	<0.0001	↑	6.02	0.017	↓	12.8	0.0007	↓	26.5	<0.0001
Connectance (CO)	1	↓	13.7	0.0002		Not retained		↑	18	<0.0001	↑	11.07	0.002		1.32	0.255
NE*NO	1		Not retained			Not retained			Not retained		↑	2.32	0.133	↓	4.1	0.047
NE*CO	1	↓	8.7	<0.0001		Not retained		↓	15	0.0003	↓	22.45	<0.0001	↓	12.7	0.0007
NO*CO	1		Not retained			Not retained		↑	5.9	0.018		Not retained			Not retained	
Model summary			*R*^2^: 0.11	AIC: 360.3		*R*^2^: 0.46,	AIC: 245.2		*R*^2^: 0.43	AIC: 229.9		*R*^2^: 0.753	AIC: −361.62		*R*^2^: 0.73	AIC: −78.90

The diversity GLM included the number of species of the resident community (continuous variable). The network GLM included bipartite network variables: nestedness, connectance, niche overlap and their first-order interactions (continuous variables). The table lists the most parsimonious models selected on the base of the AIC information coefficient via backward and forward model selection.
