# Supplementary material for: Trophic network architecture of root-associated bacterial communities determines pathogen invasion and plant health
Source: Nat Commun. 2015 Sep 24;6:8413. doi: 10.1038/ncomms9413 (PMC4598729; doi:10.1038/ncomms9413)
Supplement: Supplementary Information — Supplementary Figure 1, Supplementary Table 1-4 and Supplementary References [file ncomms9413-s1.pdf]

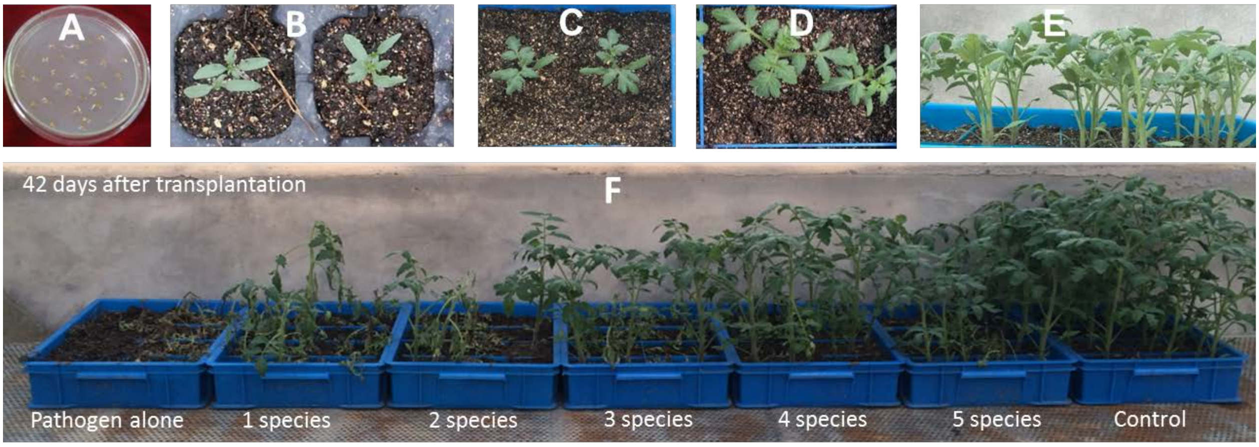

Supplementary Figure 1. Overview of the greenhouse experiment. Surface-sterilized tomato seeds (*Lycopersicon esculentum*, cultivar "Jiangshu") were first germinated on water-agar plates for 3 days (A) before sowing into seedling plates (B) containing Cobalt -60-sterilized seedling substrate (Huainong, Huaian soil and fertilizer Institute, Huaian, China). After 11 days of growth on seedling plates, tomato plants (at three-leaf stage) were transplanted to seedling trays (350mm×250mm×100mm) containing the same growth substrate as described above. Sixteen seedlings were transplanted into one seedling tray (every two plants grown in a separate cell) and trays were then subsequently inoculated with different resident bacterial communities by drenching method (final concentration of 10^{8} CFU g^{-1} soil, C; Wei *et al.* 2011). Control treatments without resident bacterial communities were also set up at this stage. Invasion experiment was initiated one week later by adding *Ralstonia solanacearum* (final concentration of 10^{6} CFU g^{-1} soil, D) to all plants. Tomato plants were grown in a greenhouse with natural temperature variation ranging from 25 °C to 35 °C and watered regularly with sterile water. The number of wilted plants per seedling plate was recorded at daily basis after 17 days post transplantation: no symptoms were observed before the day 17 (E). Invasion experiment was ended 42 days after transplantation when the control plants reached the maximal size supported by seedling trays. F) Seedling trays representative for disease incidence in plants inoculated with resident communities of increasing species richness.

Supplementary Table 1 Bacterial strains and plasmid used in this study.

Strain		Close match	GenBank No.	Source
Commensal species	QL-A2	*Ralstonia mannitolilytica*	JN699058	[1]
	QL-A3	*Ralstonia mannitolilytica*	KJ780056	[1]
	QL-A6	*Ralstonia pickettii*	HQ267096	[1]
	QL -117	*Ralstonia taiwanensis*	KJ780054	This study
	QL-140	*Ralstonia* sp.	KJ780055	This study
Pathogen	QL-Rs1115	*Ralstonia solanacearum*	GU390462	[2]
Plasmid	PYC12-M*	mCherry insert in the pYC12, under control of the constitutive Ptac promoter, gm^R		This study

*Generation of pYC12-M: The *mCherry* gene from the pTCV89 plasmid was cloned into pYC12 under the constitutive promoter P_{tac}[3]. Both plasmids were kindly donated by Associate Professor Zhong Zengtao from key lab of microbiological engineering of agricultural environment, Nanjing Agricultural University, Nanjing, China. The *mCherry* gene bordered by *Nde* I and *EcoR* I sites was amplified from plasmid pTCV89 using F1 (5’-ATG GCT CATATG GTG AGC AAG GGC GAG GAG-3’) and R1 primers (5’-GTC GAATTC AGG GCG AAT TGG AGC TCC TTA C-3’). The fragment was cloned into the plasmid pYC12 to obtain the recombinant plasmid pYC12-M. The plasmid pYC12-M was introduced into the cells of *R. solanacearum* by electroporation, transformants were selected on NAg (NA medium containing 30 μg mL^{-1} Gm) plates and fluorescence checked by fluorescence microscopy.

Supplementary Table 2 All possible community compositions of biocontrol bacterial communities

Community	QL-A2	QL-A3	QL-A6	QL-117	QL-140	Richness
Control	0	0	0	0	0	0
1	1	0	0	0	0	1
2	0	1	0	0	0	1
3	0	0	1	0	0	1
4	0	0	0	1	0	1
5	0	0	0	0	1	1
6	1	1	0	0	0	2
7	1	0	1	0	0	2
8	1	0	0	1	0	2
9	1	0	0	0	1	2
10	0	1	1	0	0	2
11	0	1	0	1	0	2
12	0	1	0	0	1	2
13	0	0	1	1	0	2
14	0	0	1	0	1	2
15	0	0	0	1	1	2
16	1	1	1	0	0	3
17	1	1	0	1	0	3
18	1	1	0	0	1	3
19	0	1	1	1	0	3
20	0	1	1	0	1	3
21	0	0	1	1	1	3
22	1	0	1	1	0	3
23	1	0	0	1	1	3
24	0	1	0	1	1	3
25	1	0	1	0	1	3
26	1	1	1	1	0	4
27	1	0	1	1	1	4
28	0	1	1	1	1	4
29	1	1	0	1	1	4
30	1	1	1	0	1	4
31	1	1	1	1	1	5

Supplementary Table 3 Classification of 48 carbons used to compose synthetic tomato exudates.

Resource name and abbreviation	Resource classification	Resource code
Acetic aicd (Ace)	organic acid	1
L-Alanine (Ala)	Amino acid	2
β-Alanine (Bala)	Amino acid	3
L-Arginine (Arg)	Amino acid	4
Ascorbic acid (Asc)	organic acid	5
L-Asparagine (Asn)	Amino acid	6
γ-Aminobutyric acid (Ami)	Amino acid	7
Citric acid (Cit)	organic acid	8
Citrulline (Cin)	Amino acid	9
Ethanolamine (Eth)	other	10
Formic acid (For)	organic acid	11
Fructose (Fruc)	sugar	12
Galacturonic acid (Galac)	organic acid	13
Glucose (Glu)	sugar	14
L-Glutamine (Gln)	Amino acid	15
Glutaric acid (Glut)	organic acid	16
L-Glycine (Gly)	Amino acid	17
Glycolic acid (Glyc)	organic acid	18
L-Histidine (His)	Amino acid	19
Isoleucine (Iso)	organic acid	20
Lactic acid (Lac)	organic acid	21
L-Lysine (Lys)	organic acid	22
L-Leucine (Leu)	organic acid	23
Maleic acid (Male)	organic acid	24
Malic acid (Mal)	organic acid	25
Malonic acid (Malon)	organic acid	26
L-Methionine (Met)	organic acid	27
Myoinositol (Mino)	other	28
2-Oxoglutaric (Oxo)	organic acid	29
L-Phenyalanine (Phe)	organic acid	30
L-Proline (Pro)	organic acid	31
Pyruvic acid (Pyr)	organic acid	32
L-Serine (Ser)	organic acid	33
Succinic acid (Succ)	organic acid	34
Sucrose (Sucr)	sugar	35
Tartaric acid (Tar)	organic acid	36
L-Threonine (Thr)	organic acid	37
L-Tryptophan (Try)	organic acid	38
L-Valine (Val)	organic acid	39
Maltose (Mal)	sugar	40
L-Arabinose (Ara)	sugar	41
D-Galactose (Gal)	sugar	42
D-Mannose (Man)	sugar	43
D-Xylose (Xyl)	sugar	44
D-Ribose (Rib)	sugar	45
D-Mannitol (Mann)	sugar	46
Inosine (Ino)	other	47

Oxalic acid(Oxa)	organic acid	48

Supplementary Table 4 Twenty different resource combinations (C1-C20) each consisting of 20 randomly selected resources. In all combinations, one indicates the presence of the given resource and zero indicates the absence of the given resource.

Resource	Resource code	C1	C2	C3	C4	C5	C6	C7	C8	C9	C10	C11	C12	C13	C14	C15	C16	C17	C18	C19	C20
Ace	1	0	0	0	1	1	0	0	1	0	1	1	1	1	1	0	0	1	1	0	1
Lala	2	1	0	1	1	0	1	1	0	1	0	0	0	0	0	1	1	0	1	1	0
Bala	3	1	1	0	0	1	0	1	1	0	0	0	0	0	1	1	1	0	1	1	0
Arg	4	0	0	1	0	1	0	0	0	0	1	1	1	1	1	0	0	1	1	1	1
Asc	5	1	1	0	0	1	1	1	0	0	0	1	1	0	0	0	1	0	1	0	0
Asn	6	0	1	1	1	0	0	1	1	0	1	0	0	1	1	0	0	1	0	0	0
Ami	7	0	0	1	1	0	0	1	0	1	1	0	0	1	0	1	0	0	0	0	1
Cit	8	1	0	0	0	1	1	0	1	1	1	1	0	0	0	0	0	0	1	1	1
Cin	9	0	1	1	0	0	0	0	1	1	1	0	1	0	1	0	1	1	0	0	1
Eth	10	0	0	0	0	0	0	0	0	0	0	0	0	0	1	0	0	1	0	0	0
For	11	0	1	0	0	0	0	1	0	1	0	1	1	0	1	1	1	0	1	0	0
Fruc	12	0	0	1	1	1	0	0	0	0	0	0	0	1	0	1	0	0	0	1	0
Galac	13	0	1	1	0	1	0	0	0	0	1	0	1	1	0	0	0	1	0	0	1
Glu	14	1	1	0	0	0	0	1	0	0	1	0	1	0	0	0	0	1	1	0	1
Gln	15	0	0	0	1	1	0	0	1	0	0	1	1	1	1	1	1	0	0	0	1
Glut	16	0	0	0	1	0	0	0	1	0	0	0	0	0	0	1	0	0	0	0	0
Gly	17	0	0	0	0	0	1	1	0	0	1	1	0	0	0	1	1	1	0	0	0
Glyc	18	1	1	0	0	0	0	0	0	0	0	0	0	0	1	1	0	0	0	1	0
His	19	0	0	0	0	1	0	0	0	1	0	0	0	0	0	1	1	1	0	0	0
Iso	20	0	0	1	0	0	1	0	1	0	1	1	0	1	1	0	0	0	1	1	0
Lac	21	0	0	1	0	1	1	1	0	0	0	0	0	1	0	1	0	0	0	0	0
Lys	22	0	0	1	1	0	1	0	0	0	0	1	0	1	0	0	1	1	1	1	1
Leu	23	0	1	0	1	0	0	0	0	0	1	1	0	1	0	0	0	0	1	0	1
Male	24	1	0	1	0	1	1	1	1	0	1	0	1	0	1	0	0	0	0	1	1

Mal	25	0	1	0	1	0	1	0	1	1	0	0	0	0	0	1	0	1	1	1	1
Malon	26	1	0	1	1	1	1	0	1	1	0	1	0	1	0	0	1	0	0	0	0
Met	27	0	1	0	0	0	0	0	1	1	0	0	0	0	0	1	1	0	0	1	1
Mino	28	1	0	0	1	0	1	0	0	0	1	1	1	0	0	0	0	1	1	1	0
Oxo	29	0	1	0	1	0	0	1	0	1	0	0	0	1	0	0	0	1	0	1	1
Phe	30	0	0	1	0	1	1	0	1	0	1	0	1	1	1	0	0	0	0	0	0
Pro	31	1	0	1	0	0	0	1	0	1	1	1	0	0	0	0	0	1	0	0	1
Pyr	32	1	1	0	0	0	1	1	0	0	1	1	0	0	1	0	0	1	1	0	0
Ser	33	1	0	0	0	0	1	0	0	0	0	1	1	0	1	1	0	0	0	0	0
Succ	34	0	0	0	1	1	1	0	1	1	1	0	1	0	0	0	1	0	1	0	0
Sucr	35	0	0	0	1	1	1	1	1	0	0	0	1	0	0	1	1	0	0	0	1
Tar	36	1	1	0	0	0	1	0	1	0	0	1	1	1	1	1	1	0	0	1	0
Thr	37	0	0	0	0	1	0	0	0	1	1	0	0	1	0	0	0	0	0	0	0
Try	38	1	1	1	1	0	0	0	1	0	0	1	0	0	1	0	0	1	1	0	0
Val	39	1	0	1	0	0	1	0	0	1	0	0	0	1	0	0	1	0	0	1	0
Mal	40	0	1	0	0	0	1	0	0	0	1	0	0	0	1	1	1	0	1	1	0
Ara	41	1	0	0	0	0	0	1	1	0	0	0	0	1	1	1	0	1	1	0	1
Gal	42	0	1	0	1	0	0	0	0	1	0	1	1	0	0	0	0	0	1	0	0
Man	43	1	0	1	1	1	0	1	0	1	0	0	1	0	0	0	1	1	0	0	0
Xyl	44	1	0	1	0	1	0	0	1	1	0	1	1	0	1	0	1	0	1	0	1
Rib	45	1	1	1	1	1	0	1	0	0	1	0	1	1	0	1	1	1	0	1	1
Mann	46	1	1	0	1	0	1	1	0	1	0	0	0	1	0	0	0	0	0	1	1
Ino	47	0	1	0	0	0	0	1	0	1	0	1	1	0	0	1	1	0	0	1	0
Oxa	48	0	0	1	0	1	0	1	1	1	0	0	0	0	1	0	0	1	0	1	0

Supplementary References:

- 1. Wei, Z. *et al*. The congeneric strain *Ralstonia pickettii* QL-A6 of *Ralstonia solanacearum* as an effective biocontrol agent for bacterial wilt of tomato. *Biological Control* **65**, 278-285 (2013).
- 2. Wei, Z. *et al*. Efficacy of Bacillus-fortified organic fertiliser in controlling bacterial wilt of tomato in the field. Appl Soil Ecol **48**, 152-159 (2011).
- 3. Wang, P., Zhong, Z. Zhou, J. Cai, T. Zhu, J. Exopolysaccharide biosynthesis is important for *Mesorhizobium tianshanense*: plant host interaction. Archives of Microbiol **189**, 525-530 (2008).
